# Advances in Understanding Salt Stress Effects on Growth and Productivity in Sorghum (*Sorghum bicolor* L. Moench)

**DOI:** 10.3390/plants15172612

**Published:** 2026-08-27

**Authors:** Xiaoqian Guo, Fadwa Bakhiet Hamid Musa, Hailu Zhu, Jianwen Zhang, Shangkun Lai, Omer Idris Musa Olom, Guisheng Zhou

**Affiliations:** 1 College for Overseas Education, Yangzhou University, Yangzhou 225009, China; 2 Jiangsu Provincial Key Laboratory of Crop Genetics and Physiology, Agricultural College, Yangzhou University, Yangzhou 225009, China; 3 Joint International Laboratory of Agriculture and Agri-Product Safety, Yangzhou University, Yangzhou 225009, China; 4 Suqian Institute of Agricultural Sciences, Jiangsu Academic of Agricultural Sciences, Suqian 223800, China; 5 Department of Forestry and Range Sciences, Faculty of Natural Resources and Environmental Studies, University of Kordofan, El Obeid 51111, Sudan

**Keywords:** sorghum, salinity stress, salt tolerance, ion homeostasis, oxidative stress, biomass, yield, breeding, PRISMA, saline soils

## Abstract

Salinity is a growing problem for cereal cultivation because it imposes multiple stresses, including osmotic, ionic, nutritional, and oxidative constraints, on the crop. Sorghum (*Sorghum bicolor* L. Moench) is considered a climate-smart C4 cereal for food, feed, fodder, forage, and bioenergy, but recent studies indicate that salinity continues to hinder establishment, biomass formation, reproductive growth, and yield. This review compiles the literature on the impacts of salinity on sorghum from 2021 to 2026, with a focus on germination, vegetative growth, physiological and biochemical responses, ion homeostasis, genetic control, productivity, mitigation, and future breeding priorities. In total, 160 records were identified, 118 records were screened after duplicate removal, and 44 recent sources were included in the synthesis. Across comparable sorghum studies, saline/NaCl treatments of approximately 60–200 mM commonly reduced germination by about 20–40%, root and shoot elongation by 25–50%, and biomass by 20–55%, while tolerant genotypes generally maintained higher K^+^/Na^+^ balance, 40–60% greater biomass retention, or two- to five-fold stronger ion homeostasis indicators than sensitive lines under similar conditions. Salt stress also lowers leaf expansion, chlorophyll stability, gas exchange, dry matter accumulation, panicle fertility, and grain filling. Tolerant genotypes show greater antioxidant potential, osmotic adjustment, photosynthetic stability, and root system resilience. Recent omics and genome-wide association studies suggest that salinity tolerance in sorghum is polygenic and involves genes related to ion transport, stress signalling, antioxidant regulation, osmolyte metabolism, and growth maintenance. This review recommends a shift from descriptive trait lists to full-cycle field validation, multi-trait selection indices, and integrated packages combining breeding with seed priming, soil water management, amendments, and beneficial microorganisms.

## 1. Introduction

Salinity is a major environmental factor affecting crop productivity in arid, semi-arid, irrigated, and coastal agricultural production systems. According to the FAO [1], salt affects more than 1381 million hectares of total land area, which is about 10.7% of the total land area of the world, and a significant part of the irrigated and rainfed cropland is already vulnerable to salinisation. Plants are injured by salinity, by low soil water potential and high concentrations of Na^+^ and Cl^−^, and through nutrient deficiency and oxidative injury [2]. Recent reviews reaffirm that this stress has a complex impact, affecting germination, root architecture, photosynthesis, hormonal pathways, antioxidant metabolism, and yield formation, which makes it challenging to manage through a single intervention [3,4,5].

In this regard, sorghum (*Sorghum bicolor* L. Moench) is a strategic crop due to its photosynthetic efficiency and adaptation to high temperature, drought, marginal soils, and low-input systems. It contributes to food security and the income of the rural population through grain, stover, forage, silage, sweet stalks, and bioenergy utilisation. Recent studies also suggest that sorghum is a climate-smart cereal and an important model crop for monocot stress biology due to its genetic versatility, physiology, and root characteristics; thus, it can be studied in different environments [6,7]. Sorghum growth decreases under moderate and high salinity [8], especially in situations where establishment and leaf area are limited by salinity, which also reduces photosynthetic efficiency and ion balance and disrupts grain filling [9,10].

In recent years, sorghum-specific studies have demonstrated that salt tolerance is highly genotypic. Under saline stress, some genotypes maintain better germination, chlorophyll stability, photosynthesis, Na^+^ exclusion and K^+^ retention, osmolyte accumulation and antioxidant activity, while sensitive lines show early membrane damage, disruption of nutrients and loss of dry matter [11,12,13]. In addition, molecular studies have evolved from just phenotypic screening to the discovery of candidate genes, comparative transcriptomics and genome-wide association analysis (GWAS) [14,15,16]. These developments indicate that salinity tolerance in sorghum is a complex phenotype involving establishment, root-zone adaptation, ion regulation, redox control, and reproductive stability.

Notwithstanding this progress, the literature is still disjointed. Most studies focus on germination, plant characteristics/salinity under controlled conditions, or plant responses to salinity; fewer focus on germination and plant responses to salinity followed by plant reproductive response, forage quality, grain yield, or management packages of interest to the farmer. This review should, thus, not merely state that salinity is a problem; it should determine whether there is ample evidence to confirm salinity as a problem, disagreements in studies, the most useful traits for breeding, and the ability to use field management techniques to turn physiological tolerance into productivity. This review is based on the existing systematic review of the literature in the past five years, based on evidence from 2021 to 2026. It aims to (i) summarise the effects of salinity on sorghum throughout the crop’s life; (ii) integrate physiological, biochemical, ionic and molecular mechanisms; (iii) compare salinity indicators at the genotype level; (iv) assess management options; and (v) suggest research priorities to enable the development of productive sorghum systems in salt-affected soils.

Accordingly, this paper offers an updated perspective, focusing on productivity as the primary criterion while restricting its mechanistic analysis to factors that explain agronomic performance.

## 2. Review Methodology and Search Counts

A systematic review with narrative synthesis was conducted using PRISMA 2020-informed screening principles to improve transparency [17] while retaining the flexibility required for a mechanistic crop science synthesis [18]. This manuscript is, therefore, intended to be handled as a systematic review with narrative synthesis rather than a meta-analysis. The review question was as follows: how does salinity affect sorghum growth and productivity, and which traits or interventions most consistently support tolerance? The search was completed on 22 June 2026 and focused on the literature published from 2021 to 2026 so that the evidence base reflected recent plant science, agronomy, molecular breeding and salinity management developments. The search combined publisher databases, PubMed, Google Scholar-indexed records, open journal platforms, and reference list chasing. The search strings included sorghum salinity stress, *Sorghum bicolor* salt tolerance, sorghum germination salinity, sorghum Na/K salinity, sweet sorghum salt stress gene expression, sorghum salinity biomass yield, salinity stress crop plants review, biochar salinity stress plants, PGPR salinity stress and seed priming sorghum salt stress.

**Figure 1:**
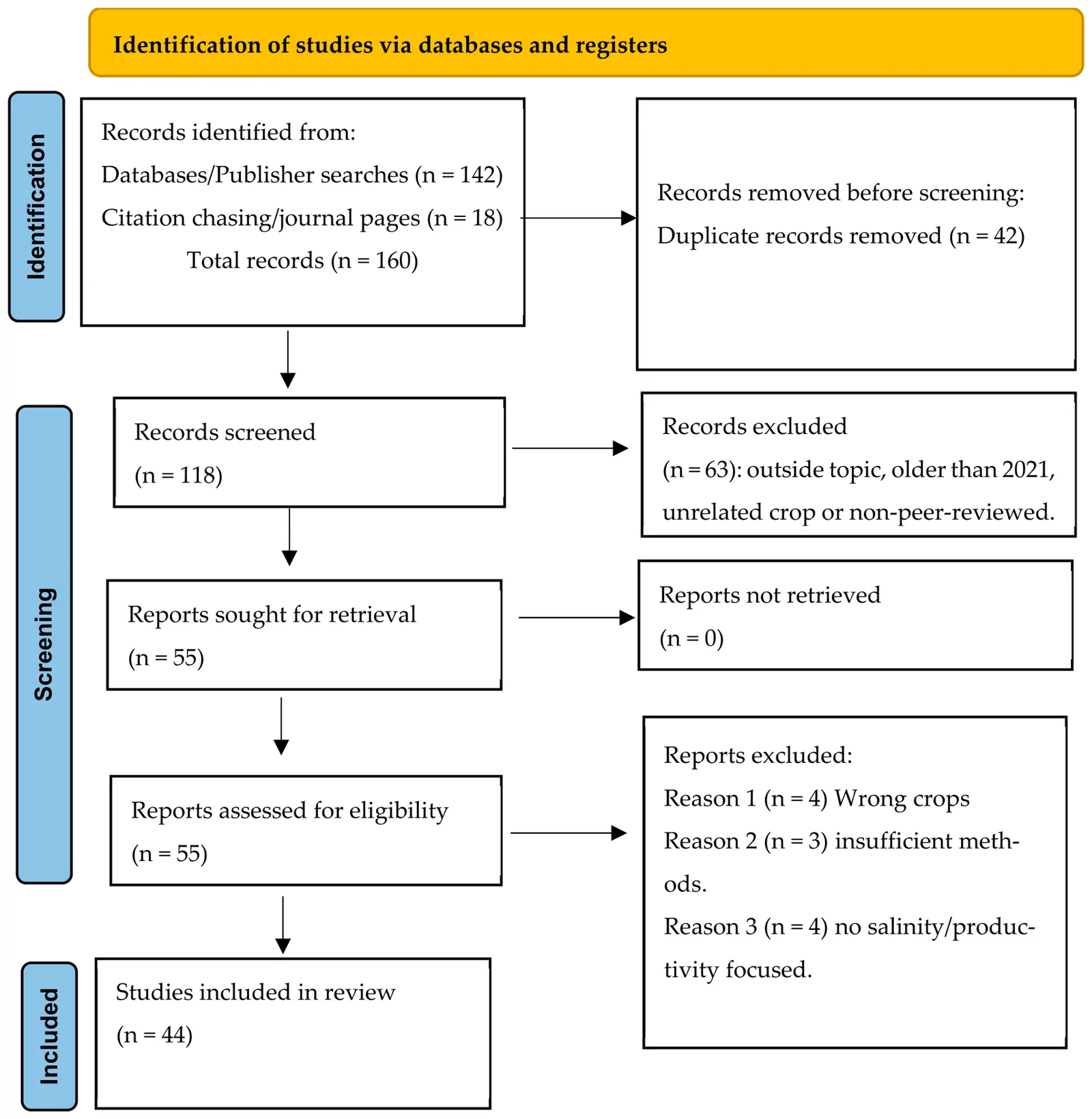
PRISMA-style literature selection flow with search counts for the 2021–2026 evidence corpus.

A total of 142 records were found by searching databases and publishers, and 18 additional records were found by citation chasing and journal pages, giving 160 records before duplicate removal. Duplicate removal eliminated 42 records, leaving 118 records for title and abstract screening. Sixty-three records were excluded at this stage for being outside the scope of the topic, older than the 2021–2026 inclusion window, focused on unrelated crops without transferable salinity mechanisms, or non-peer-reviewed. Fifty-five full texts were then assessed. Eleven full texts were excluded because they focused on the wrong crop without relevant salinity mechanisms (4), lacked sufficient methodological detail (2), did not focus on salinity–productivity links (3), or duplicated data already covered in a more complete article (2). Forty-four recent sources were included in the final qualitative synthesis. These counts are reported in the revised PRISMA flow diagram (Figure 1), which illustrates the number of studies that went from identification to inclusion [19]. Two reviewer-recommended contextual references were added during revision to strengthen molecular, plant–microbe and AI breeding discussions; these were not counted in the original PRISMA corpus.

Articles were included if they were peer-reviewed, review articles, field experiments, greenhouse experiments, laboratory experiments, molecular studies, or authoritative reports in the last 6 years (2021 to 2026) and focused on sorghum, salinity, plant stress physiology, soil management, seed priming, PGPR, biochar, or breeding, as applicable. Studies that measured growth, germination, root traits, photosynthesis, ion balance, oxidative stress, osmolytes, biomass, yield, gene expression, GWAS, QTLs, or management outcomes were prioritised. The exclusion criteria were non-scientific sources; duplicate records; inaccessible and/or incomplete bibliographic information; studies that discussed only unrelated stresses; and studies that lacked a direct connection with growth or productivity. The type of synthesis was thematic, rather than meta-analytic, because salinity units, genotypes, growth stages, experimental systems, and outcome variables showed significant differences among studies.

For each included source, the evidence type, salinity type, stress concentration or EC where reported, stress duration, growth stage, study environment, genotype or crop material, main trait, and tolerance implication were coded. This coding enabled comparisons between early-stage screening papers, whole-plant physiology studies, field-oriented biomass/yield papers, management studies, and mechanistic molecular studies. The synthesis emphasises convergent evidence, but it also flags cases where controlled NaCl or pot findings require field validation. The full 44-source coding matrix is provided as Supplementary Table S1.

A structured quality appraisal procedure was applied to the empirical and directly relevant management studies. Each study was assessed on four criteria: (1) clarity of the salinity treatment or soil diagnosis, (2) relevance of measured traits to growth or productivity, (3) adequacy of genotype or management description, and (4) linkage between mechanisms and agronomic performance. Each criterion was scored as 0 = absent, 1 = partially reported, or 2 = clearly reported, giving a maximum qualitative appraisal score of 8. Studies with weak salinity descriptions or without growth/productivity outcomes were used only as contextual evidence. A formal meta-analysis was not conducted because salinity units, genotypes, growth stages, experimental systems, and outcome variables differed substantially among studies.

The coded evidence profile showed that the corpus is weighted toward controlled experimentation. Of the 44 included sources, approximately 19 were controlled laboratory, pot, greenhouse or omics studies, 5 were field-based or field-oriented sorghum/agronomic studies, and 20 were broader reviews, soil status reports, management papers or methodological sources. Therefore, conclusions on NaCl response, early-stage screening, ion homeostasis and candidate genes are relatively well supported, whereas conclusions on farmer field productivity, sodic soils and long-term integrated packages require further validation.

## 3. Soil Salinity and Sorghum Relevance in Salt-Affected Agriculture

**Figure 2:**
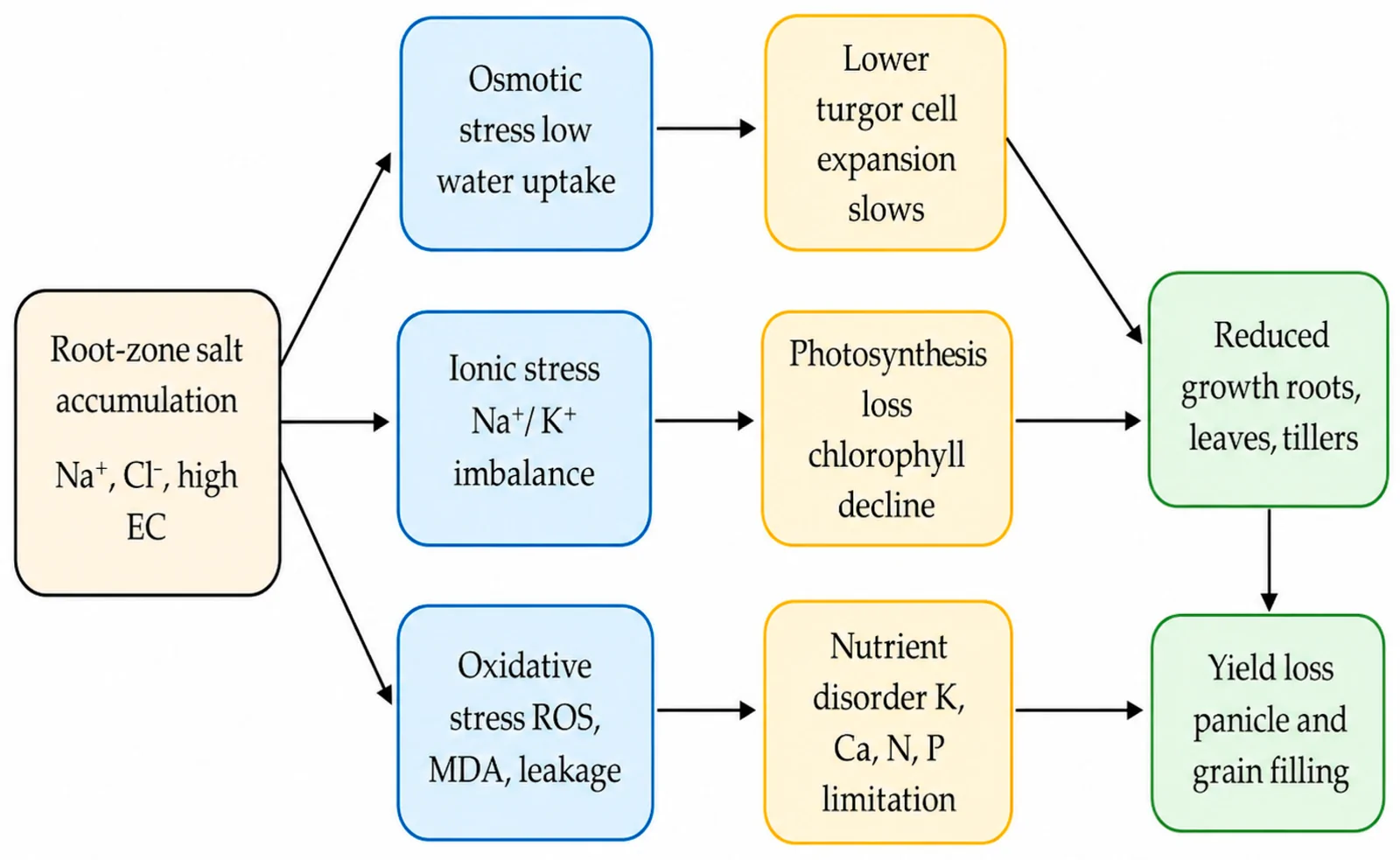
Mechanistic pathway from root-zone salinity to sorghum growth and yield loss.

Soils are considered agronomically saline when soluble salts in the root zone interfere with plant water absorption or cause toxic ionic concentrations in the soil (Figure 2). In many salinised systems, sodium and chloride are the major ions present, but sulphate, carbonate, bicarbonate, calcium, and magnesium are also important in influencing the electrical conductivity of the soil and the classification of soils as saline, sodic, or saline–sodic. Poor drainage, saline irrigation water, rising groundwater levels, high evaporation, or intrusion of seawater are especially detrimental in areas where salinity is affecting the land. The FAO [1] assessment points out that there is still a lot of uncertainty in certain areas, but the main trend is that salinity is a growing problem, as climate change, water scarcity, and more intensive irrigation pressures on soils are increasing.

The initial response of the plant is through osmotic stress. If the salt concentration outside the roots is high, the water potential in the soil around the roots is reduced, and the seed, seedlings, and mature roots are unable to take up water as readily as when the soil is moist. This physiological drought inhibits cell growth, leaf emergence, and root growth. A secondary phase occurs when Na^+^ and Cl^−^ subsequently build up in plant tissues, which affects the activity of enzymes, membrane selectivity, the movement of nutrients, and chloroplast function. Recent salinity evidence also identifies oxidative stress as an interacting component. Oxidative stress occurs when oxidative stimuli such as ROS attack lipids, proteins, nucleic acids, and pigments, leading to oxidative stress when antioxidant systems are overwhelmed [3,4,20].

The relevance of sorghum is that it is already grown in stressed areas, and its many applications make it appealing for marginal lands (Table 1). The crop can be used for grain (food), stover and forage (animal feed), and sweet stalks (bioenergy), as well as for biomass (silage). Such versatility lowers risk in vulnerable farming systems; biomass can also be used for feed if grain yield decreases, and phenology and C4 metabolism can be used to ensure system survival during seasonal stress. However, it is these same qualities of sorghum that are under threat from salinity. Grain, forage, and bioenergy productivity can all be reduced simultaneously due to reduced root length, smaller leaf size, decreased chlorophyll, weak photosynthesis, and poor panicle development [10,12].

One key finding from recent research is that sorghum should not be categorised simply as salt-tolerant in an absolute sense, but rather as salt-responsive. It is genotype-specific, relative, and stage-dependent in terms of its level of tolerance. Some of the genotypes can grow in 60–120 mM NaCl or moderate EC soil, and others have significant reductions in their germination, dry weight, ion balance and photosynthetic performance [9,13]. Therefore, sorghum is a useful crop for salt-affected agriculture provided genotype screening, soil water management, and field validation are viewed as interdependent components of the same production system.

However, the usefulness of sorghum in saline situations is not a foregone conclusion. It is C4-efficient, drought-resistant, and water-use-efficient, but salinity is a limiting factor that is distinct from drought. Salinity has two components—water limitation and ion toxicity and antagonism—while drought is primarily water limitation. This difference is important for breeding because it is possible that a drought-resistant genotype could effectively save water but accumulate toxic sodium ions or lose potassium under saline irrigation conditions. Improvement of sorghum for drought, ion homeostasis and root-zone adaptation should, therefore, be integrated, particularly in regions of overlap between dryland salinity and coastal intrusion, and in regions where a poor-quality irrigation source is used.

### Salinity Type, Sodicity and Soil Diagnosis

The reviewed evidence does not support treating all salt-affected soils as one uniform stress. Saline soils primarily impose soluble-salt osmotic stress and Na^+^/Cl^−^ toxicity, whereas sodic soils involve high exchangeable sodium, poor aggregation, low infiltration, surface crusting and restricted root growth. Saline–sodic soils combine both problems, so management requires both salt leaching and correction of sodicity. In the 44-source corpus, most sorghum-specific experimental studies addressed saline or NaCl-based stress; sodic and saline–sodic conditions were represented mainly by the literature on soil reclamation and management. No included sorghum genotype study directly ranked the same genotypes across saline, sodic and saline–sodic soils. Consequently, genotype-ranking conclusions in this review apply most strongly to saline/NaCl conditions, while recommendations involving gypsum and reclamation apply primarily to sodic or saline–sodic soils.

## 4. Evidence from Germination and Seedling Establishment

**Figure 3:**
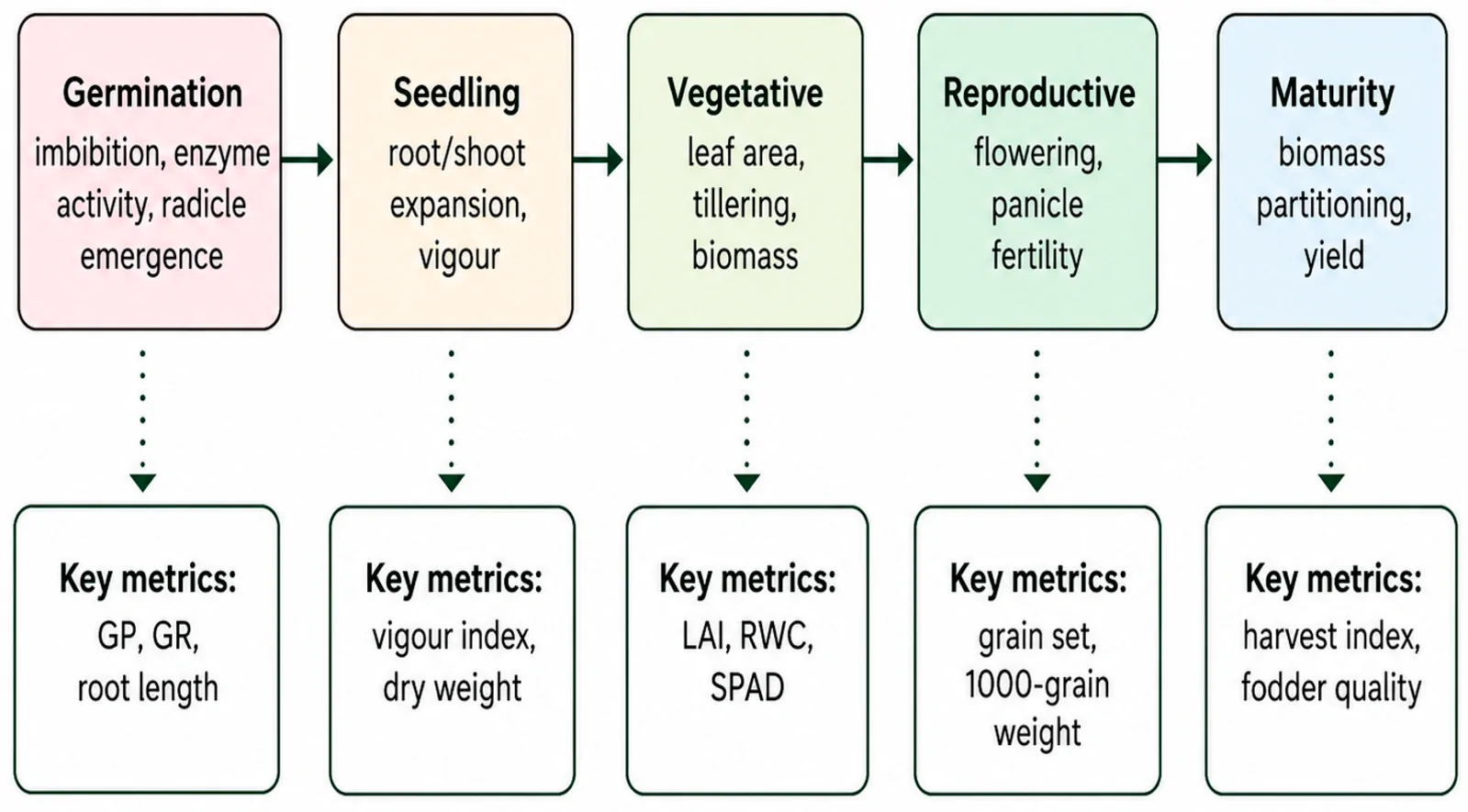
Growth-stage sensitivity of sorghum to salinity and key traits for screening.

Germination and seedling establishment are among the most frequently studied stages in sorghum because they provide clear screening endpoints and strongly influence final stand density (Figure 3). Salinity affects germination by limiting imbibition, enzyme activation and reserve mobilisation. In the reviewed early-stage studies, 100 and 200 mM NaCl significantly decreased the germination percentage, germination rate, root length, shoot length and biomass of seven sorghum varieties, with Debuday showing relatively better performance [21]. Across comparable NaCl-based seedling studies, effect-size ranges commonly showed about 20–40% reductions in germination and 25–50% reductions in root and shoot elongation relative to controls. Salt-sensitive cultivars experienced larger reductions in seedling growth and vigour, whereas tolerant cultivars maintained stronger roots, shoots and membrane stability under the same stress [9,16].

At the seedling stage, the root is particularly significant as it is the first organ that is in contact with the saline medium and will dictate the subsequent access to water and nutrients. Salt stress may cause a reduction in primary root length, decrease in lateral root formation, changes in meristem organisation and interference with vascular differentiation. A salt stress tolerance trait is early root architecture, which was established as a growth trait by Peduzzi et al. [22], who also demonstrated that early root architecture is a mechanistic tolerance trait through alteration of root meristem definition, vascular differentiation and the metabolome. Good root system development enables plants to access a deeper soil volume and may enhance recovery from transient salinity; however, in severe salinity, meristem activity is suppressed, and the root/shoot system is reduced, resulting in reduced ability to establish in the field.

One of the drawbacks is that early-stage tolerance is not necessarily a good predictor of final yield. Germination characteristics might help detect vigorous lines, but reproductive salinity can also cause a decrease in panicle fertility and, consequently, in grain filling and the harvest index. Future screening for breeding relevance should link germination percentage, seedling vigour, root characteristics, and ion balance with emergence in the field, biomass partitioning, and yield of grain or forage. The most helpful early traits are, therefore, those that are predictive of performance across environments; for example, seedling dry weight, root length, Na^+^/K^+^ ratio, and membrane stability and recovery after stress relief [12,16].

The most robust message from seedling studies is that salinity tolerance at this stage is multidimensional. A salt-tolerant seedling needs to absorb sufficient water to germinate, to maintain the function of the membrane, to regulate the early uptake of ions, to protect the meristems of the roots, and to initiate the mobilisation of reserves. It is, thus, less informative to report only the percentage of germination than to report the percentage of germination including root length, shoot length, dry weight, Na^+^, K^+^, and oxidative stress indicators. Root characteristics are especially relevant for breeding, since they will determine the crop’s ability to avoid salt accumulation at the surface, to extract water from the subsoil, and to sustain nutrient uptake. Early screening should, thus, be retained but modified as a portal for complete cycle tolerance assessment.

### Temporal Dynamics, Stress Timing and Recovery Capacity

Salinity should also be interpreted as a dynamic stress rather than a fixed treatment (Table 2). Acute salinity, such as sudden exposure to NaCl in hydroponic or germination assays, tends to reveal rapid osmotic injury, membrane leakage and reduced elongation. Chronic field salinity develops more gradually and can combine osmotic stress, ion accumulation, nutrient imbalance and soil structure constraints. Timing is equally important: exposure during emergence reduces stand density; exposure during vegetative growth lowers leaf area and biomass; exposure during flowering reduces panicle fertility; and exposure during grain filling reduces grain weight and harvest index. Recovery after rainfall, leaching or improved irrigation depends on whether root meristems, photosynthetic tissues and K^+^/Na^+^ balance remain functional. Sequential stresses are particularly important in drylands because salinity may be followed by heat or drought, while chronic salinity can accumulate across seasons if salts are not leached below the root zone.

## 5. Phenotypic, Physiological, and Biochemical Responses

A reduction in vegetative growth is a perceptible sign of underlying physiological stress. Impacts of salinity include plants that are shorter, fewer in number, and smaller in leaf area, fewer tillers, smaller stems, a smaller canopy, lower fresh weight, and lower dry weight. These changes are due to restricted cell growth, decreased turgidity, impairment of nutrient uptake, and carbon fixation. In forage sorghum, vegetative biomass is directly valuable, and thus a loss of leaf area, tillers, and stems is a physiological as well as an economic loss. Dewi et al. [10] demonstrated that both salinity and drought–salinity interactions reduced biomass and grain yield in tropical sorghum varieties, thereby demonstrating that whole-plant productivity was dependent on vegetative function under combined field stresses.

Photosynthesis is one of the key processes that is sensitive to salinity. Water saving due to salt-induced stomatal closure is accompanied by reduced CO_2_ uptake, and non-stomatal limitations include damage to chloroplasts, loss of pigment, impaired electron transport, and increased production of ROS. Amombo et al. [13] evaluated forage sorghum varieties and determined that traits related to salt performance can be identified at various growth stages using photosynthetic regulation. Their findings are significant because they are not only survival traits but are also linked to photosynthetic performance and forage productivity. When assessing salinity tolerance, it is, therefore, recommended that chlorophyll content, stomatal conductance, Fv/Fm, performance index, and gas exchange also be measured, in addition to morphological measurements.

Biochemical changes are also observed as an additional indicator of tolerance vs sensitivity. An increase in salinity causes reactive oxygen species, hydrogen peroxide, lipid peroxidation, and electrolyte leakage to occur [26]. Tolerant sorghum lines usually increase antioxidant enzyme activity (superoxide dismutase, catalase, peroxidase, and ascorbate peroxidase), reduce malondialdehyde levels, and increase membrane stability. Punia et al. [11,27] correlated the responses of sorghum seedlings to saline conditions with reserve mobilisation, antioxidant potential, metabolome adjustment and ascorbate–glutathione scavenging. Mulaudzi et al. [28] also reported that chitosan controlled oxidative stress and antioxidant activity under salt stress in sorghum, highlighting the need for redox regulation [29].

Another common tolerance marker is compatible solutes. Proline, soluble sugars, glycine betaine, and other osmolytes are involved in maintaining cellular water and protein stability and protecting membranes. But it is important to be cautious when interpreting osmolytes. Typically, positive proline is thought to be a sign of adaptive osmotic adjustment for tolerant genotypes, whereas it may also be a reflection of the severity of injury in sensitive ones. Sagar et al. [12] demonstrated that salt tolerance in sorghum was not merely attributed to proline levels but also to osmoprotectant regulation, sodium extrusion, and photosynthetic efficiency. It helps to interpret the results of this multi-trait approach by combining osmolyte accumulation with growth, ion balance, photosynthesis, and membrane stability data.

The most important physiological finding is that tolerance is a performance phenotype, rather than a stress response phenotype (Table 3). Photosynthesis, water relations, and reproductive development may fail despite a plant’s attempts to increase antioxidants or proline, resulting in loss of productivity. Thus, integrated sets of traits, such as relative water content, SPAD/chlorophyll, stomatal conductance, photosynthetic rate, Na^+^/K^+^ ratio, MDA, electrolyte leakage, antioxidant enzymes, root dry weight, biomass, and yield, are good candidates for evidence. These comprehensive indicators provide a rationale for the maintenance of productive function in some sorghum lines and for their survival under salinity [9,23].

Another critical aspect of this is the difference between stress avoidance and stress tolerance. There are some genotypes that not only limit leaf area and stomatal conductance but also do so strongly, which results in a lower water loss rate and a reduction in carbon gain. This approach can provide a short-term survival benefit, but it could reduce biomass and grain production. Other genotypes have moderate gas exchange, stable chlorophyll and water status, and biomass continues to accumulate. The latter is more useful for agriculture. Therefore, consideration of physiological measurements needs to relate to productivity, not just to stress intensity. A genotype with reduced stomatal conductance will not necessarily be tolerant; it will be tolerant only if its reduced stomatal conductance does not result in a negative water balance with an unacceptably low carbon assimilation and reproductive output.

Homeostasis of ions is a key aspect of salinity tolerance in sorghum. Under saline conditions, roots take in water from a solution with a high concentration of Na^+^ and Cl^−^. High levels of sodium compete with potassium at uptake and transport sites, and high levels of chloride can disrupt photosynthesis and nitrogen metabolism. A lower K^+^/Na^+^ ratio is closely correlated with poor physiological performance, as potassium plays important roles in stomatal regulation, protein synthesis, activation of enzymes, and transport of carbohydrates and osmotic adjustment. Several recent studies have found that tolerant sorghum lines retain more potassium (K^+^) and accumulate less sodium (Na^+^) in sensitive tissues or have higher K^+^/Na^+^ ratios than sensitive lines [9,12,21].

Also important is the distribution of sodium in the roots, stems, and leaves. Tolerant genotypes can decrease the loading of Na^+^ in the xylem, retain more Na^+^ in the roots, create vacuoles for the storage of excess ions, or sequester ions in older tissues. This decreases the toxicity in young leaves, chloroplasts, and actively growing meristems. However, ion transport cannot be divorced from transcriptional regulation, as suggested in the study by Kang et al. [15], which compared the growth of cultivars of sweet sorghum under NaCl treatments and correlated ion accumulation with gene expression. Likewise, Wang et al. [16] found genomic regions and candidate genes associated with germination salt tolerance, such as ion transport, stress signalling and growth regulation genes.

Calcium, magnesium, nitrogen, and phosphorus are also affected by salinity. Calcium stabilises membranes and is important for signalling; magnesium is central to chlorophyll; and nitrogen and phosphorus are central to protein synthesis, energy transfer and biomass formation. Osmotic stress decreases mass flow to roots; high sodium decreases calcium availability and affects the uptake of nutrients. The reason that salinity symptoms frequently involve chlorosis, poor root growth, reduced photosynthesis, and low dry matter is due to these changes. In wider crop reviews, it is highlighted that nutrient imbalance is not a secondary injurious mechanism, but one of the three primary ones, along with osmotic and ionic toxicity [3,4].

## 6. Molecular and Omics Evidence

**Figure 4:**
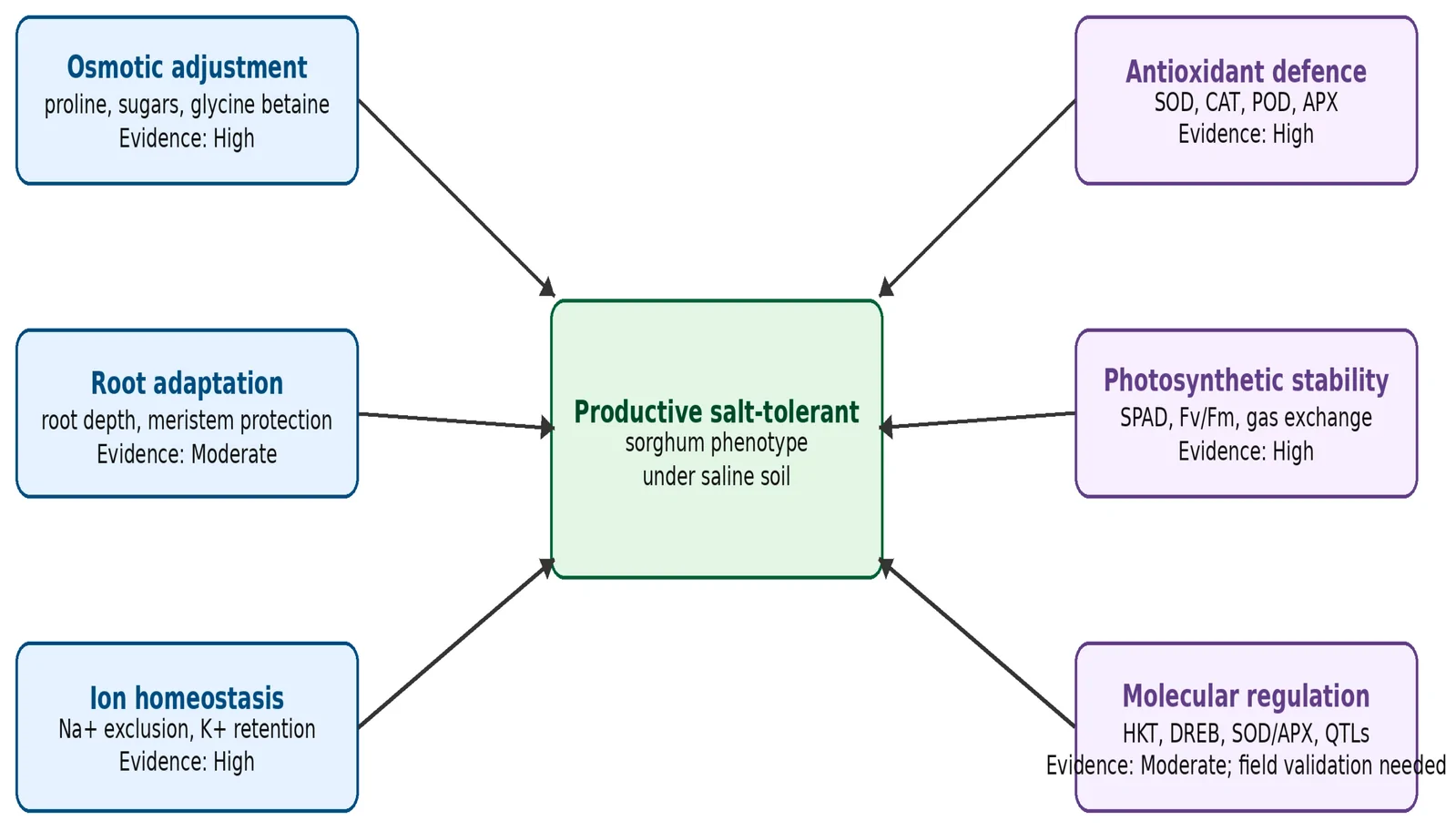
Integrated tolerance mechanism model for sorghum under salinity stress with pathway confidence ratings.

There has been a recent increase in molecular studies that support the scientific research on sorghum salinity (Figure 4). Comparative transcriptomic analysis was performed to identify candidate genes and non-synonymous SNPs linked to salt tolerance between a salt-tolerant mutant and the wild type, including candidate genes that were associated with salt tolerance, including some that may be related to stress metabolism and dhurrin-related pathways. Wang et al. [16] performed a GWAS on 245 mini-core accessions and millions of SNPs, identifying 35 salt-tolerant loci and 39 salt-tolerant candidate genes during germination. The results suggest that salt tolerance is a polygenic trait and that there may be untapped genetic potential for the marker-assisted breeding of sorghum.

Gene expression analysis reveals that tolerance is associated with pathways of ion transporters, antioxidants, dehydration sensors, osmolyte pathways, and stress signalling networks. Alzahrani et al. [23] assessed the drought, salinity, and combined stress responses of sorghum genotypes and found variations in their physiological, biochemical, and expression responses, such as genes encoding SbSOD, SbAPX, SbCAT, SbHKT, SbDREB, and SbDHN. These outcomes are important as they consider a combination of stresses rather than salinity alone, which is more representative of dryland systems than salinity alone. They also reveal that the genotype may respond differently when subjected to a single stress compared with multiple stresses, so single-stress screening is not enough for climate-resilient agriculture.

Sweet sorghum research has introduced another level: the interaction of salinity and sugar content, source–sink relationships, and energy characteristics. Kang et al. [15] investigated the differences between sweet sorghum cultivars through physiological and gene expression analyses, and Sun et al. [30] analysed the photosynthetic efficiency, biomass and sugar accumulation of cultivars in response to combined water and salt stress. These studies imply that salinity studies cannot be conducted with all sorghum types as the same species. The mechanisms of tolerance are likely shared between grain sorghum, forage sorghum, and sweet sorghum, but the selections for grain yield, biomass for forage, sugar content, or dual-purpose stability are different, as is the trait weighting.

The omics evidence is good but not yet fully manifested in field-ready cultivars. There are numerous candidate genes yet to be functionally validated, and GWAS loci need to be evaluated in multiple environments, developmental stages, and genetic backgrounds. Quality phenotyping is also required for molecular breeding. In such cases, if salinity in the field is not measured correctly or if yield traits are not available, the genomic associations will help identify the stress response genes that may not have a positive effect on yield. The most helpful future strategy will, thus, be to integrate phenomics and genomics and measure root architecture, photosynthesis, ion balance, antioxidant response, biomass, and yield while, at the same time, mapping markers and expression profiles. This would increase the realism of sorghum salinity breeding in terms of its applicability and mechanistic depth [5,31]. Recent crop-level genomic and biotechnological syntheses further support this interpretation by emphasising functional validation, discovery of novel salt tolerance genes, genomic selection, and the need to connect molecular candidates with field performance [32].

Trait architecture is another aspect that must be considered when translating omics to breeding. Apparently, many genes with small-to-moderate effects control salinity tolerance, which affects root growth, ion transport, osmotic adjustment, ROS scavenging, and flowering stability. This polygenic architecture is conducive to genomic selection and multi-environment prediction, not one marker. When traits like Na^+^/K^+^ regulation or germination under salinity are easily measured, however, marker-assisted selection is still valuable when loci have consistent effects on these traits. Therefore, a combination of high-throughput phenotyping, genomic prediction, and targeted testing of candidate genes in elite breeding backgrounds may serve as the optimum approach.

## 7. Biomass, Reproductive Development, and Yield Productivity

The most important measurement of salinity tolerance is productivity. Many studies show that there are reductions in germination or in seedling growth; the value of the plant to the farmer is related to biomass, grain yield, fodder yield, or silage and sweet-stalk yield. The effects of salinity on biomass include the following: limitation in root growth, leaf size and growth, photosynthetic area, nutrient uptake, and assimilate production. Lower aboveground biomass has an immediate impact on the quantity of green fodder, hay, and silage. Grain sorghum vegetative source capacity is reduced, which restricts panicle development and grain filling. The links explain that biomass traits are not just simple growth traits but incorporate stress history and that they can predict yield potential in later growth stages [10,13,33].

Across the comparable sorghum-focused studies, response magnitudes were strongly treatment- and genotype-dependent. Moderate to severe saline treatments, commonly in the range of 60–200 mM NaCl or equivalent elevated EC, generally reduced germination by about 20–40%, root and shoot elongation by 25–50%, and whole-plant biomass by 20–55% relative to controls. In the limited reproductive-stage evidence, tolerant genotypes retained approximately 40–60% higher biomass or grain-related performance than sensitive genotypes, but these estimates remain more robust for controlled saline conditions than for sodic or saline–sodic field soils.

The development of reproduction is frequently more yield-sensitive than early development. Although stress before flowering does not seem to be detrimental to panicle fertility and grain weight, before-flowering stress can reduce panicle initiation and vigour, and stress during flowering can decrease pollen viability, grain set, and panicle fertility, and stress during grain filling can decrease harvest index and grain weight. This is a critical problem for sorghum, as often terminal drought and heat are coupled with salinity in marginal environments. As photosynthesis decreases and leaves age too quickly, less of the assimilate is transferred to grain or to maintaining the quality of the stover. Research on effects of drought–salinity interactions in tropical sorghum indicates that biomass and grain yield can decrease simultaneously, and responses to water-use efficiency vary by cultivar [10].

Tolerant lines appear to have coordinated traits that allow them to stay productive, according to genotypic evidence. Sagar et al. [12] found hybrid sorgo to be a good performer in saline conditions as it retained morphological traits, photosynthetic efficiency, K^+^/Na^+^ balance and osmoprotectant regulation. Rajabi Dehnavi et al. [9] found variation among ten genotypes in the absence and presence of 60 and 120 mM NaCl; salinity had different impacts on biomass and physiological traits for the lines. Under pot salinity, Haque et al. [34] also reported that variation in morpho-physiological and biochemical traits occurred at the reproductive stage. These studies indicate that the following traits are needed in a high-yielding tolerant genotype: early establishment and reproductive stability.

The important thing to remember is that productivity must not be assumed based on a single characteristic. A genotype with high vigour (seedling) may not flower, a genotype with high vegetative biomass may not allocate resources efficiently to grain, and a genotype with high antioxidant activity may not yield due to low photosynthesis. However, the following full-cycle trait sets and data should be used in the evaluation: salinity level, soil EC, water quality, genotype identity, growth stage, biomass, grain yields, forage traits, and ion data. These details make the studies difficult to compare and can only have limited breeding implications without them.

When grown as a dual-purpose crop, sorghum presents an added difficulty in evaluation because grain and biomass responses could be different. A line with a high level of salinity tolerance could have a low rate of grain filling, and a line with a high level of grain filling could have low forage production under livestock systems. Therefore, the type of production should be mentioned in the studies. In salt-affected drylands, the most resilient cultivar might be one that can produce grain yields that are satisfactory while also providing usable stover or forage when grain yield is lower. This risk-buffering value is usually not included in traditional yield tests but is very relevant in smallholder systems where livestock depend on crop residues for feed during shortages.

## 8. Agronomic and Biological Management Strategies

**Figure 5:**
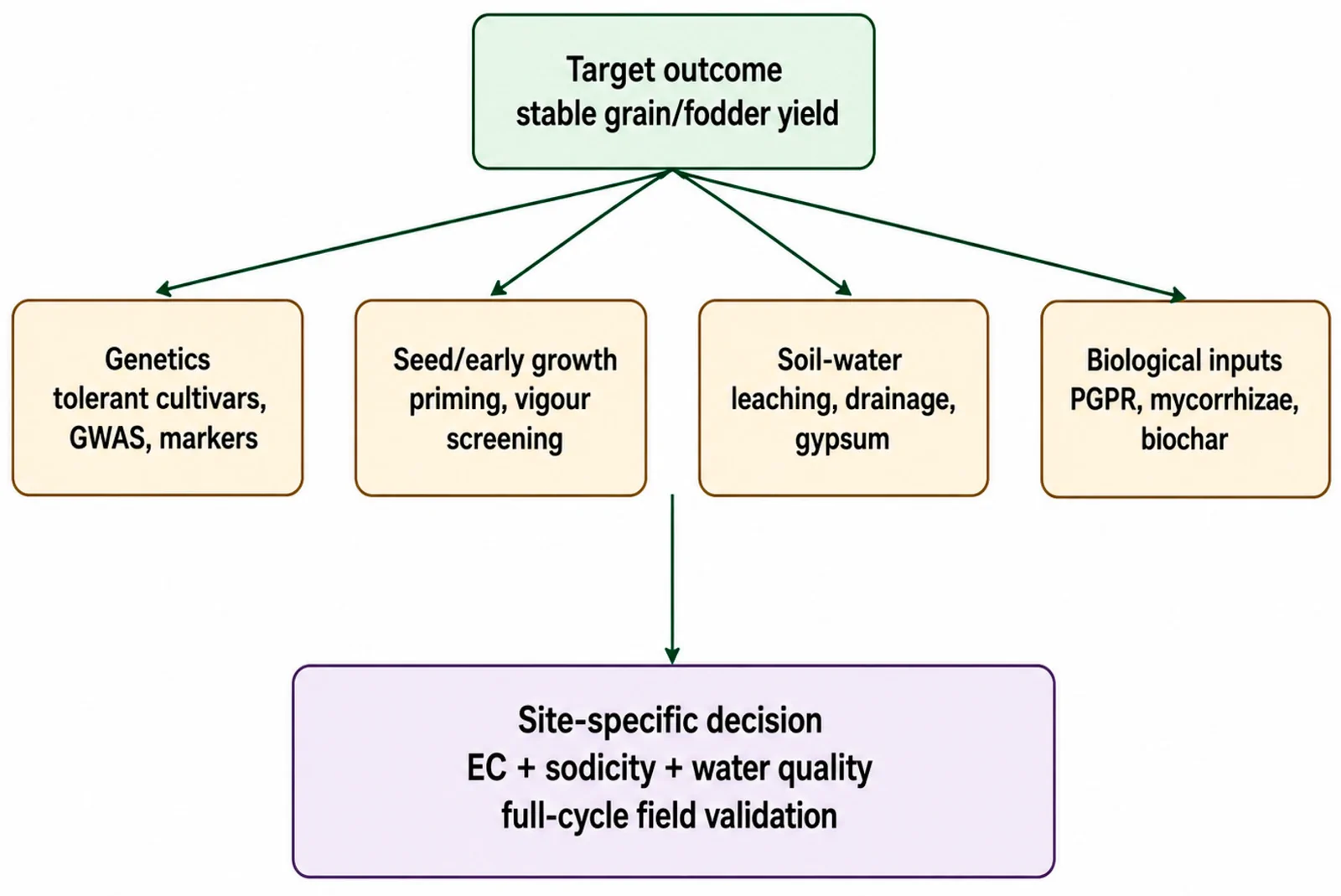
Field-ready framework for improving sorghum productivity in saline soils.

Integrated management is needed for sustainable sorghum production in saline soils since salinity is a soil–water–plant problem [35]. While a cultivar is the basis of tolerance to salt, poor drainage, high root-zone EC, sodicity, nutrient imbalance, or low seedling establishment cannot be overcome with salt-tolerant cultivars. According to the FAO [1], an integrated approach to managing salt-affected soils should include crop selection, drainage, and leaching, soil amendments, and improved water management. In the case of sorghum, this involves aligning the genotype tolerance with irrigation quality, soil EC, sodicity status, and farmer resources and target use: grain, forage, sweet sorghum, or dual-purpose production (Figure 5).

Seed priming is directly relevant as salinity frequently causes injury to sorghum before canopy establishment [36]. In this regard, Guo et al. [24] reported that priming enhanced growth of sorghum under salt stress by enhancing antioxidative defence, and Hassan et al. [25] reported that zinc seed priming helped reduce salt stress effects on germination, physiological and biochemical functions of sorghum. Priming can improve emergence uniformity, seedling vigour, and early biomass but should be evaluated at the local level as the effect of priming agents varies with seed lot, genotype, salinity level, and may interact with each other.

Soil amendments are another useful practice. Organic amendments are able to raise organic matter, cation exchange capacity, microbial activity, and water-holding capacity. Biochar has emerged as an increasingly popular soil amendment due to its ability to improve soil structure, decrease Na^+^ and Cl^−^ uptake, increase the growth of roots, promote chlorophyll production, and alter the regulation of osmolyte/hormones under salinity [37,38,39]. Gypsum is most beneficial in sodic or saline–sodic soils because calcium will displace the exchangeable sodium ion, which will improve aggregation, infiltration, and leaching. But not all saline soils are suitable for treatment with gypsum; gypsum would work on sodicity, drainage and adequate water for leaching [1,40].

Biological methods involve plant-growth-promoting rhizobacteria, mycorrhizae, and other beneficial microbes [41]. Recent reviews have identified PGPR as potential salinity alleviation agents as they improve nutrient uptake, modulate hormones, produce osmolytes, produce ACC deaminase activity, and stimulate exopolysaccharide production and antioxidant activity [42,43]. For cereals, these mechanisms are promising, but their effects in the field are not always consistent, as microbial effects are dependent on the soil, the level of salinity, the climate, and the crop genotype. When considering microbial interventions for sorghum, biomass and yield endpoints should be considered, not seedling or pot traits. This is consistent with recent plant–microbe interaction frameworks showing that rhizosphere benefits depend on microbial community composition, interaction pathways, host genotype, soil conditions and targeted microbiome application strategies [44].

Therefore, the management synthesis supports a package approach as follows: tolerant cultivar selection; seed priming for establishment (if necessary); organic amendment or biochar, if soil condition needs improvement, and only when recommended by a local expert; balanced fertilisation to correct soil nutrient imbalance; improved irrigation scheduling and drainage to minimise salt accumulation in the crop root zone; and microbial inoculants when locally validated. The most significant contribution is not to describe these interventions but to perceive them as decision points related to soil diagnosis and crop target.

There is also a difference between saline constraints and sodic constraints in the management recommendations. The first issue in saline soils is soluble salt in the root zone, and the issues of leaching, drainage, irrigation management, and crop tolerance are of central importance. In sodic soils, reduction in infiltration and restriction of root growth are more important than soil structure, and calcium sources such as gypsum are more relevant. Salinity and sodicity are often used interchangeably in many papers; precision in diagnosis is necessary. Leaching can be ineffective if sodicity is not corrected and the soil is saline (salty); the application of gypsum to a saline but not sodic soil may add costs but not address the primary issue of sodicity.

Therefore, the management synthesis supports a package approach as follows: tolerant cultivar selection; seed priming for establishment (if necessary); organic amendment or biochar, if soil condition needs improvement, and only when recommended by a local expert; balanced fertilisation to correct soil nutrient imbalance; improved irrigation scheduling and drainage to minimise salt accumulation in the crop root zone; and microbial inoculants when locally validated (Table 4). The best contribution is not to just mention these interventions but to also see them as decision points related to soil diagnosis and crop target.

## 9. Breeding and Screening Implications

Multi-stage and multi-trait selection is needed in sorghum salinity tolerance breeding. Early screening can identify genotypes with high germination, seedling vigour, root growth and initial ion regulation, but further screening is required for vegetative biomass, reproductive stability and yield (Table 5). The recent GWAS by Wang et al. [16], the transcriptomic study by Jeon et al. [14], and the combined-stress analysis by Alzahrani et al. [23] and broader advances in AI-enabled genomic prediction [45] indicate that molecular tools can assist selection only when they are linked to field phenotyping, soil EC and productivity outcomes. Useful markers are those that will enhance establishment and productivity under saline soil field conditions.

The following breeding indices should be recommended for a practical breeding index: germination percentage, germination speed, root length, seedling dry weight, relative water content, chlorophyll/SPAD, stomatal conductance, photosynthetic rate, K^+^/Na^+^, MDA/electrolyte leakage, antioxidant enzyme activity, biomass retention, panicle fertility, grain number, grain weight, harvest index, and forage quality. Some traits may not require measurement in each breeding cycle, but key traits should be as standardised as possible for comparison. This is not the case yet and is one reason why it has been hard to synthesise the literature across genotypes and environments.

Product-specific breeding targets should also be used for sorghum. Grain sorghum requires yield stability and grain filling; forage sorghum requires biomass retention, leaf proportion, and feed quality; and sweet sorghum requires stalk biomass, sugar content, and stress-stable photosynthesis. According to Sun et al. [30] and Kang et al. [15], sugar metabolism and growth processes of sweet sorghum need to be taken into account. Considering all sorghum types as a single ideotype could mask differences in salinity tolerance and farmer value.

Last but not least, combined stress should be considered when breeding. In most saline areas, salinity is accompanied by drought, high temperatures, and nutrient deficiency or waterlogging. Overall, the combined-stress results suggest that genotype rank can shift under salinity conditions when drought is combined with salinity [10,23]. Thus, screening for the next generation should be factorial and/or field-natural combinations and not just NaCl treatments alone. This change would bring breeding outcomes closer to reality and make sorghum breeding more resilient in a changing climate.

The process could be made more efficient with high-throughput phenotyping and AI-assisted genomic prediction. Screening large panels with chlorophyll fluorescence imaging, spectral indices, automated root phenotyping and canopy temperature measurements can precede detailed destructive sampling, while AI-driven genomic prediction can help integrate marker, phenotypic and environmental information for multi-environment selection [46]. However, technology must enhance biological interpretation rather than replace it. Remote and image-based traits should be calibrated against ion composition, biomass and yield under clearly defined salinity conditions. Poorly calibrated screens may identify green-looking plants that are not productive, whereas well-calibrated pipelines can identify genotypes that sustain physiological function, biomass and reproductive stability across stages and environments.

## 10. Research Gaps and Future Directions

The first big gap is the disparity between controlled-environment studies and field testing. Laboratory and greenhouse studies are useful for isolating mechanisms but tend to employ sudden exposure to NaCl, which may not reflect the gradual process of salinisation in the field, and heterogeneous soils or variable irrigation water. Soil EC, if applicable, and sodium adsorption ratio, irrigation water quality, growth stage, climate conditions, and yield endpoints should be reported in future studies. Without these details, mechanistic results are difficult to extrapolate to field management.

The second is the lack of a link between molecular discoveries and productivity. Many more candidate genes, SNPs, QTLs, and expression profiles are now available, though not validated across genotypes and environments. Functional validation and marker development/field selection must be linked. For traits like HKT-mediated ion transport, antioxidant gene expression, osmolyte pathways, and stress-responsive transcription factors, which can confer growth advantages under certain conditions but growth costs under others, such traits are particularly important [5,14,16].

The third gap is in relation to the management packages. There is promising evidence for the use of seed priming, biochar, organic amendments, gypsum, PGPR, and improved irrigation, although few experiments have been conducted with combinations in sorghum field systems under economic conditions. Farmer adoption relies on cost, labour, availability of local materials, water availability, and market target. Integrated packages should be compared with each other in different saline environments, and the grain yield, forage yield, soil improvement, and input cost and risk reduction should be quantified for future research.

The last one is review-level synthesis. It is common to find papers on salinity in crops or on individual traits in sorghum in the existing literature. This review points to the need for a growing degree-day-based sorghum-specific framework based on growth stage, mechanism, genotype, and management. The use of a registered systematic procedure, exported data from the Scopus and Web of Science databases, quantitative meta-analysis if comparable data are available, and open additional data with a screening matrix would be beneficial.

Finally, open evidence matrices are being developed. Future reviews and breeding programmes would benefit if authors could publish salinity level, genotype, growth stage, soil or solution conditions, measured traits, statistical outcomes, and raw data or supplementary data in reusable formats. This would enable meta-analysis of the effect sizes for biomass, germination, and yield, and enable comparisons of the reliability of the trait across studies. The literature is abundant, but it is not always consistent. There are many useful results reported, but with a lack of environmental description or incompatible units. Standardisation of reporting would improve the quality of sorghum salinity science and enhance the defensibility of future submissions [47].

## 11. Conclusions

Salinity reduces sorghum growth and productivity through osmotic water limitation, ion toxicity, nutrient imbalance, oxidative stress and impaired photosynthesis. Across comparable studies, saline/NaCl treatments of approximately 60–200 mM generally reduced germination by about 20–40%, root and shoot elongation by 25–50%, and biomass by 20–55% compared with controls. These estimates are strongest for saline/NaCl and controlled-environment studies; evidence for sodic and saline–sodic field soils is less direct and must be interpreted through soil diagnosis, sodicity status and drainage conditions.

The strongest tolerance evidence supports integrated traits rather than single markers. Productive tolerant genotypes maintain root function, K^+^/Na^+^ balance, chlorophyll stability, gas exchange, antioxidant protection, osmotic adjustment and biomass partitioning. Tolerant lines often maintain 40–60% higher biomass retention or two- to five-fold-stronger ion homeostasis indicators than sensitive lines under similar saline treatments, but only a small proportion of the reviewed evidence is field-based. Therefore, genotype rankings from hydroponic, germination or pot assays should be treated as provisional until confirmed under target saline, sodic or saline–sodic field conditions.

Management conclusions also depend on salinity type and duration. In saline soils, priority should be given to tolerant cultivars, leaching, drainage, irrigation quality control and balanced nutrition. In sodic soils, calcium amendment and structure repair should precede leaching. In saline–sodic soils, the sequencing of gypsum, leaching and drainage is critical. Seed priming, biochar, organic amendments and PGPR can support establishment and stress buffering, but their value depends on local soil chemistry, input cost, labour availability, water resources and whether the production target is grain, forage, sweet-stalk biomass or dual-purpose sorghum.

Future priorities should, therefore, move beyond stress assays toward field-validated breeding management pipelines. Research should quantify effect sizes, separate acute from chronic salinity, measure recovery capacity after rainfall or leaching, compare genotype rankings across saline and sodic conditions, and combine omics, phenomics, AI-driven genomic prediction and farmer-oriented management trials. This would make sorghum salinity research more useful for improving establishment, biomass production, grain stability and sustainable production in salt-affected agricultural systems.

## Figures and Tables

**Table 1:** Recent sorghum salinity studies with high relevance to the synthesis.

Study	Salinity Type/Environment	Type of Evidence	Key Contribution to This Review
[21]	Saline/NaCl; laboratory germination	Sorghum germination experiment	NaCl reduced germination, root/shoot length, and biomass; variety differences supported early-stage screening.
[9]	Saline/NaCl; controlled genotype comparison	Sorghum genotype comparison	Ten genotypes exposed to 60 and 120 mM NaCl showed divergent biomass, physiological, and biochemical responses.
[13]	Saline/controlled forage sorghum physiology	Forage sorghum photosynthesis study	Photosynthetic regulation and PIABS helped identify salt performance traits linked to forage yield.
[22]	Saline/NaCl; root anatomy and metabolome	Root anatomy/metabolome	Salt stress altered root meristem definition, vascular differentiation, and metabolome.
[12]	Saline/NaCl; morpho-physiological screen	Morpho-physiological genotype screen	Photosynthetic efficiency, sodium extrusion, and osmoprotectants explained tolerance in hybrid sorghum.
[14]	Saline/molecular screening	Comparative transcriptomics	Candidate salt tolerance genes and SNPs were identified in a mutant sorghum line.
[16]	Saline/germination-stage GWAS	GWAS	A total of 245 mini-core accessions produced 35 loci and 39 candidate genes for germination-stage salt tolerance.
[23]	Combined salinity and drought; controlled stress	Combined drought-salinity study	Physiology, antioxidants, and stress gene expression varied across genotypes under individual and combined stresses.
[24]	Saline/NaCl plus seed priming	Seed-priming experiment	Priming improved salt-stressed sorghum growth through antioxidative defence.
[25]	Saline/NaCl plus zinc priming	Zinc-seed-priming experiment	Zinc priming improved sorghum germination, growth, and biochemical functioning under salinity.

**Table 2:** Temporal dynamics of salinity stress in sorghum.

Temporal Condition	Main Response Expected	Key Measurements	Interpretive Value
Acute early exposure	Rapid osmotic inhibition and reduced root/shoot elongation	GP, GR, root length, and electrolyte leakage	Useful for rapid screening but not sufficient for yield prediction.
Chronic vegetative exposure	Progressive ion accumulation, chlorophyll decline and biomass loss	SPAD, RWC, Na^+^/K^+^, and dry matter	Better predictor of forage potential and source capacity.
Flowering-stage exposure	Lower panicle fertility and grain set	Panicle fertility, grain number, and pollen viability	Critical for judging reproductive tolerance.
Grain-filling exposure	Reduced grain weight and harvest index	1000-grain weight and harvest index	Links stress duration to final productivity.
Recovery period after leaching/rainfall	Partial restoration if meristems and leaves remain functional	New leaf growth, root recovery, and K^+^/Na^+^ balance	Separates reversible stress from irreversible injury.
Sequential salinity-drought/heat	Rank changes among genotypes	Combined-stress biomass/yield and phenology	Needed for realistic dryland breeding.

**Table 3:** Physiological and biochemical markers of sorghum salinity tolerance.

Tolerance Mechanism	Representative Markers	Breeding/Management Implication
Osmotic adjustment	Proline, soluble sugars, glycine betaine, and leaf water status	Supports water balance; interpret with growth and yield to avoid mistaking injury response for tolerance.
Ion homeostasis	Na^+^, K^+^, K^+^/Na^+^, Ca^2+^, and tissue ion partitioning	Key for selection; tolerant lines preserve K^+^ and restrict toxic Na^+^ in young leaves.
Antioxidant defence	SOD, CAT, POD, APX, MDA, and electrolyte leakage	Lower injury and higher detoxification indicate cellular protection.
Root adaptation	Root length, root dry weight, meristem integrity, and root-to-shoot ratio	Improves water/nutrient acquisition and early establishment.
Molecular regulation	HKT, DREB, SOD/APX/CAT genes, GWAS loci, and SNP markers	Requires functional validation and field phenotyping before breeding deployment.

**Table 4:** Integrated management decision matrix for sorghum productivity in salt-affected soils.

Soil Diagnosis	Recommended Sequence and Suitable Strategies	Cautions, Contraindications and Adoption Issues
Saline soil (high EC; low sodicity)	Select tolerant cultivar -> improve irrigation scheduling -> leach salts where water and drainage allow. Use seed priming, balanced nutrition, drainage and organic amendments as supporting strategies.	Gypsum alone is not a primary solution where sodicity is absent. Water availability, drainage infrastructure and labour determine feasibility.
Sodic soil (high exchangeable Na; poor structure)	Diagnose sodicity -> apply calcium amendment if recommended -> improve infiltration -> leach sodium -> establish tolerant cultivar after structure improves.	Leaching without correcting sodicity may fail because infiltration remains poor. Gypsum cost, transport, application rate and soil-testing capacity are key constraints.
Saline–sodic soil	Correct sodicity first -> provide drainage -> leach soluble salts -> establish tolerant cultivar. Combine gypsum before leaching with organic amendments, drainage and seed priming where appropriate.	Unsequenced leaching or over-irrigation can worsen waterlogging and salt redistribution. Requires coordinated water, amendment and labour resources.
Saline irrigation water	Assess water EC/SAR -> schedule irrigation -> blend or alternate water if possible -> monitor root-zone EC.	Repeated poor-quality irrigation without leaching causes cumulative salinity. Monitoring and alternative water sources may be unavailable to smallholders.
Uncertain diagnosis	Test soil EC, pH, and SAR/ESP before intervention; seek local extension advice.	Avoid recommending gypsum, heavy fertiliser or microbial inputs without diagnosis. Low-cost diagnosis prevents unnecessary input expenditure.
Irrigation and drainage	High conceptually.	Reduces root-zone salt accumulation.

**Table 5:** Trait utility ranking for sorghum salinity breeding and screening.

Trait Set	Cost/Throughput	Predictive Value for Yield	Best Use	Redundancy or Caution	Trait Set
Germination %, speed and seedling vigour	Low cost/high throughput	Moderate; useful for stand establishment	Initial screening of large panels	Does not guarantee reproductive tolerance.	Germination %, speed and seedling vigour
Root length, root dry weight and root architecture	Moderate/medium throughput	High for establishment and recovery	Detailed breeding evaluation	Root traits must be linked to biomass and ion data.	Root length, root dry weight and root architecture
SPAD, chlorophyll fluorescence and gas exchange	Moderate/medium–high throughput	High when calibrated to bio-mass or forage yield	Physiological selection and stress monitoring	Green leaves can reflect survival but not necessarily yield.	SPAD, chlorophyll fluorescence and gas exchange
Na^+^, K^+^ and K^+^/Na^+^ ratio	Moderate cost/medium throughput	High for saline NaCl conditions	Ion homeostasis selection	Tissue and growth stage must be standardized.	Na^+^, K^+^ and K^+^/Na^+^ ratio
Antioxidant enzymes (SOD, CAT, POD, and APX) and MDA	Higher cost/lower throughput	Moderate; explains injury response	Mechanistic validation	Enzyme assays may be redundant and should be reduced to representative markers.	Antioxidant enzymes (SOD, CAT, POD, and APX) and MDA
Biomass, panicle fertility, grain weight and harvest index	Moderate–high cost/lower throughput	Very high for productivity	Final field validation	Requires field or full-cycle controlled validation.	Biomass, panicle fertility, grain weight and harvest index

## Data Availability

No new data were created or analysed in this study. Data sharing is not applicable to this article.

## Supplementary Materials

The following supporting information can be downloaded at https://www.mdpi.com/article/10.3390/plants15172612/s1, Table S1: Evidence matrix for study comparability and field validity.
