# Supplementary material for: Advances in Understanding Salt Stress Effects on Growth and Productivity in Sorghum (Sorghum bicolor L. Moench)
Source: Plants (Basel). 2026 Aug 27;15(17):2612. doi: 10.3390/plants15172612 (PMC13566990; doi:10.3390/plants15172612)
Supplement: Supplementary file 1 [file plants-15-02612-s001.zip › plants-4500539-supplementary.pdf]

Supplementary Material Table S1. Evidence matrix for study comparability and field validity

This matrix was added in response to reviewer requests to show coding by salinity type, environment, growth stage, and study purpose and field validity. The first 44 entries correspond to the PRISMA-counted synthesis corpus; entries 32, 44 and 46 are reviewer-recommended contextual references added during revision and are not included in the original screening count.

Ref.	Source category	Salinity type	Environment/evidence	Stage/trait focus	Field validity
**1**	Soil-status report	Saline/sodic/saline-sodic	Global assessment	Soil extent and diagnosis	High for soil context
**2**	Review	General salinity	Crop physiology	Hormones/signalling	Contextual
**3**	Review	General salinity	Crop physiology	Mechanisms/management	Contextual
**4**	Review	General salinity	Crop genetics	Genetic/molecular approaches	Contextual
**5**	Review	General salinity	Crop molecular biology	Adaptive mechanisms	Contextual
**6**	Crop overview	Not applicable	Institutional crop profile	Sorghum importance	Contextual
**7**	Review/model crop	Drought/salinity context	Crop review	Sorghum stress biology	Contextual
**8**	Sorghum study	Saline irrigation	Likely field/pot	Growth and alleviation	Moderate; needs site detail
**9**	Sorghum synthesis/study	NaCl/saline	Controlled genotype comparison	Physiology/biochemistry	Moderate-high for saline traits

**10**	Sorghum study	Drought + salinity	Controlled/pot or field-oriented	Biomass/yield physiology	Useful for combined stress
**11**	Sorghum seedling study	NaCl	Laboratory/controlled	Reserve mobilisation/antioxidants	Early-stage evidence
**12**	Sorghum genotype study	Saline/NaCl	Controlled genotype screen	Photosynthesis/Na extrusion	High for traits; field validation needed
**13**	Forage sorghum study	Saline/NaCl	Controlled/greenhouse	Photosynthetic performance	High for forage physiology
**14**	Sorghum transcriptomics	Salt/NaCl	Molecular dataset	Candidate genes/SNPs	Needs functional validation
**15**	Sweet sorghum study	NaCl	Controlled physiology/gene expression	Sweet sorghum ion/gene response	Moderate
**16**	Sorghum GWAS	NaCl germination	Genomic panel	Germination-stage salt tolerance	Strong genomic screening; field validation needed
**17**	Review methods	Not applicable	Methodology	Review search/synthesis	Methodological
**18**	Narrative review methods	Not applicable	Methodology	Narrative synthesis	Methodological
**19**	PRISMA guideline	Not applicable	Reporting guideline	Systematic review reporting	Methodological
**20**	Review	General salinity	Plant physiology	Salt-resistance mechanisms	Contextual

**21**	Sorghum germination study	NaCl	Laboratory/controlled	Germination/seedling growth	Early-stage evidence
**22**	Sorghum root study	Salt stress	Controlled/anatomy/metabolome	Root meristem/metabolome	Mechanistic
**23**	Sorghum combined stress study	Salinity + drought	Controlled genotype study	Physiology/gene expression	Useful for combined stress
**24**	Sorghum priming study	NaCl	Controlled experiment	Seed priming/antioxidants	Management support
**25**	Sorghum priming study	Salinity	Controlled experiment	Zinc priming/growth	Management support
**26**	Review	Abiotic stress	Plant physiology	ROS and antioxidant defence	Contextual
**27**	Sorghum metabolome study	NaCl	Controlled/omics	Ascorbate-glutathione/metabolome	Mechanistic
**28**	Sorghum chitosan study	Salinity	Controlled experiment	Antioxidant response	Management/biochemical
**29**	Sorghum stress study	Salt	Controlled experiment	Proline/antioxidant genes	Mechanistic
**30**	Sweet sorghum study	Water + salt	Controlled/field-oriented	Photosynthesis/biomass/sugar	Production-type specific

**31**	Review	General salinity	Breeding review	Tolerance mechanisms/breeding	Contextual
**32**	Cereal study	Salt stress	Experimental cereal comparison	Agronomic/yield traits	Transferable but not sorghum-specific
**33**	Sorghum pot study	Salinity	Pot culture	Reproductive morpho-physiology	Useful controlled productivity evidence
**34**	Review	Salinity/sodicity	Agronomy review	Field crop management	High for management context
**35**	Sorghum seed/molecular study	Stress/salinity	Experimental/molecular	Emergence/resilience	Emerging evidence
**36**	Review	Salinity	Biochar review	Soil amendment mechanisms	Management context
**37**	Review	Abiotic stress/salinity	Biochar review	Crop traits/soil functioning	Management context
**38**	Review	Soil salinity	Biochar review	Salinity remediation	Management context
**39**	Review	Salinity/sodicity	Soil amendment review	Rehabilitation	Management context
**40**	Review	Abiotic stress	PGPR review	Microbial resilience	Management context
**41**	Review	Salinity stress	PGPR review	Crop productivity	Management context

**42**	Review	Rice salinity	PGPR review	Microbial mechanisms	Transferable microbial context
**43**	Review	Abiotic stress	Biotechnology review	Sorghum resilience	Contextual
**44**	Review	Saline/sodic	Soil reclamation	Reclamation advances	Management context
**45**	Reviewer-added review	General salinity	Genomics/biotechnology	Salt-tolerance genes/precision breeding	Contextual, added post-review
**46**	Reviewer-added review	Rhizosphere interactions	Microbiome review	Plant-microbe mechanisms	Contextual, added post-review
**47**	Reviewer-added perspective	Not salinity-specific	AI/genomic prediction	AI breeding/genomic prediction	Contextual, added post-review
